# Supplementary material for: Recovery after human bone marrow mesenchymal stem cells (hBM-MSCs)-derived extracellular vesicles (EVs) treatment in post-MCAO rats requires repeated handling
Source: PLoS One. 2024 Oct 21;19(10):e0312298. doi: 10.1371/journal.pone.0312298 (PMC11493303; doi:10.1371/journal.pone.0312298)
Supplement: S1 File — (PDF) [file pone.0312298.s001.pdf]

SUPPLEMENTARY FIGURES

Recovery after human bone marrow mesenchymal stem cells (hBM-MSCs)-derived extracellular vesicles (EVs) treatment in post-MCAO rats requires repeated handling.

Figure S1. All animals recover their weight loss following MCAO surgery after 28 days post stroke (dps), independently of treatment

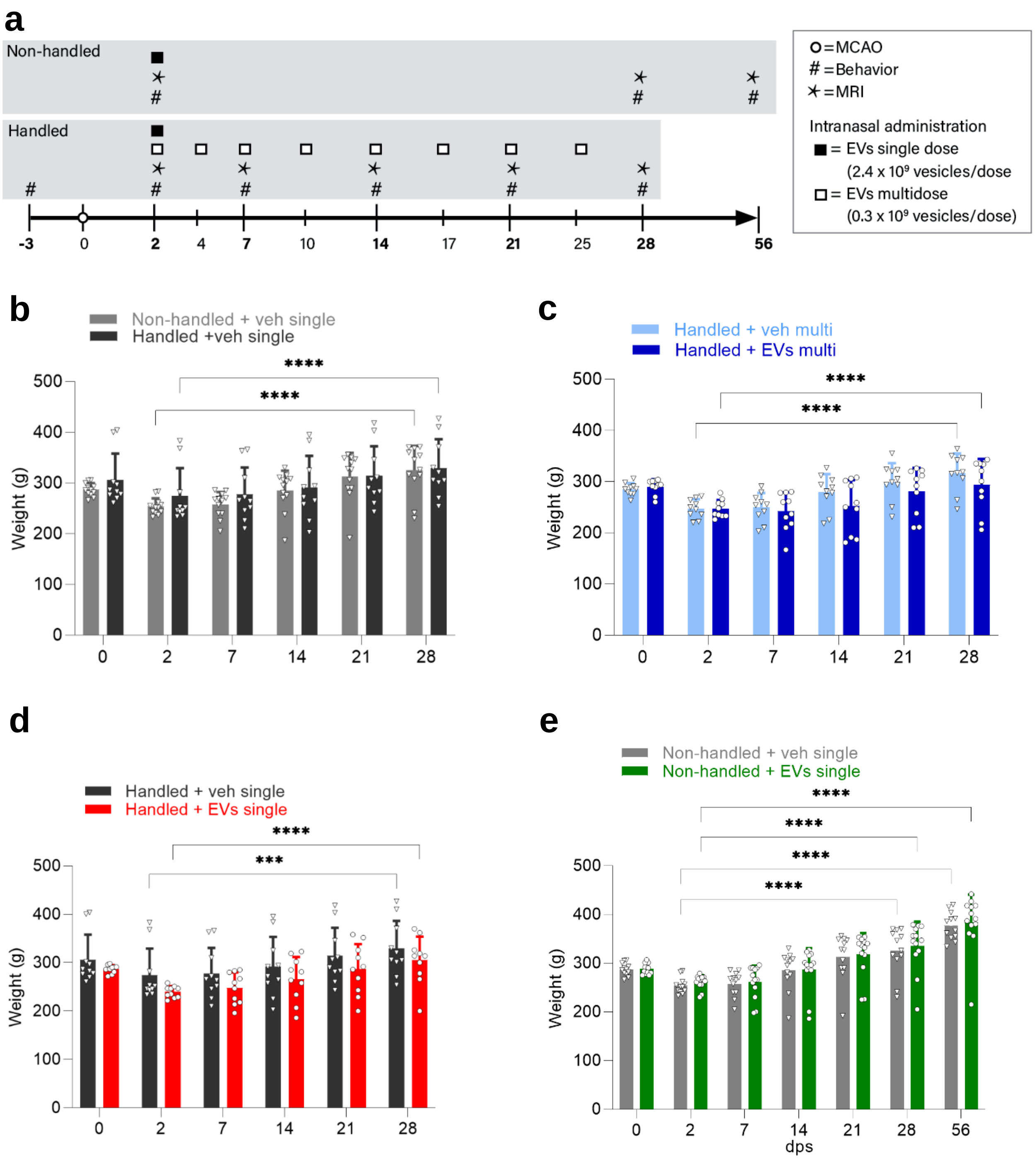

Figure S1. All animals recover their weight loss following MCAO surgery after 28 days post stroke (dps), independently of treatment.
(a) Experimental design and timeline with the different cohorts and treatments. The exact number of adult MCAO rats used in each cohort and their treatment is specified in **Table S1**.
(b) Weights (g) of non-handled and handled rats, receiving a single intranasal dose of vehicle, at 0, 2, 7, 14, 21, and 28 dps.
(c) Weights (g) of handled rats, receiving a multidose of vehicle or EVs, at 0, 2, 7, 14, 21, and 28 dps.
(d) Weights (g) of handled rats, receiving a single dose of vehicle or EVs, at 0, 2, 7, 14, 21, and 28 dps.
(e) Weights (g) of non-handled rats, receiving a single dose of vehicle or EVs, at 0, 2, 7, 14, 21, 28, and 56 dps.
All data was analyzed by two-way repeated measures ANOVA followed by the post hoc Holm-Sidak's multiple comparisons test (***$p<0.0005$, ****$p<0.0001$). Bars show mean +/− SD. Each symbol represents a single rat: non-handled + veh single (*n=12*), non-handled + EVs single (*n=13*), handled + veh multi (*n=10*), handled + EVs multi (*n=10*), handled + veh single (*n=10*), handled + EVs single (*n=10*).

Figure S2. Animals improve spontaneously the total ischemic volume and percentage of edematous expansion of the ipsilateral hemisphere at 28 dps, independently of the treatment.

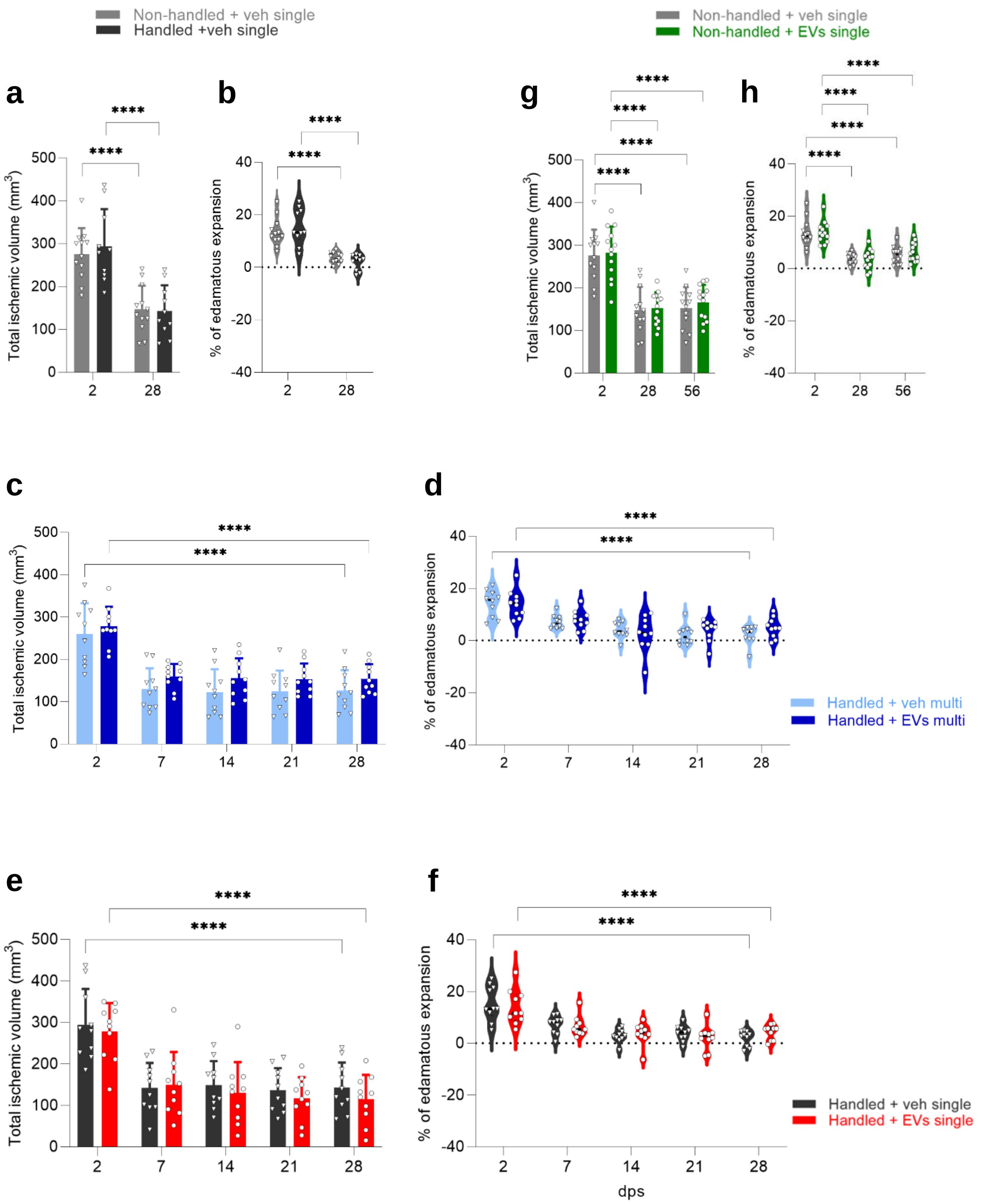

Figure S2. Animals improve spontaneously the total ischemic volume and percentage of edematous expansion of the ipsilateral hemisphere at 28 dps, independently of the treatment.

(a–b) Total ischemic volume and percentage of edematous expansion of the ipsilateral hemisphere in non-handled and handled rats, receiving a single intranasal dose of vehicle, at 2 and 28 dps.

(c–d) Total ischemic volume and percentage of edematous expansion of the ipsilateral hemisphere in handled rats, receiving a multidose of vehicle or EVs, at 2, 7, 14, 21, and 28 dps.

(e–f) Total ischemic volume and percentage of edematous expansion of the ipsilateral hemisphere in handled rats, receiving a single dose of vehicle or EVs, at 2, 7, 14, 21, and 28 dps.

(g–h) Total ischemic volume and percentage of edematous expansion of the ipsilateral hemisphere in non-handled rats, receiving a single dose of vehicle or EVs, at 2, 28, and 56 dps

All data was analyzed by two-way repeated measures ANOVA followed by the post hoc Holm-Sidak's multiple comparisons test (****$p<0.0001$). Bars show mean +/− SD. Violin plot graphs represent quartiles and median. Each symbol represents a single rat: non-handled + veh single (*n=12*), non-handled + EVs single (*n=13*), handled + veh multi (*n=10*), handled + EVs multi (*n=10*), handled + veh single (*n=10*), handled + EVs single (*n=10*).

Figure S3. Handled rats treated with multidose EVs do not recover in the cylinder, grid walking, or forelimb placing tests at 28 dps

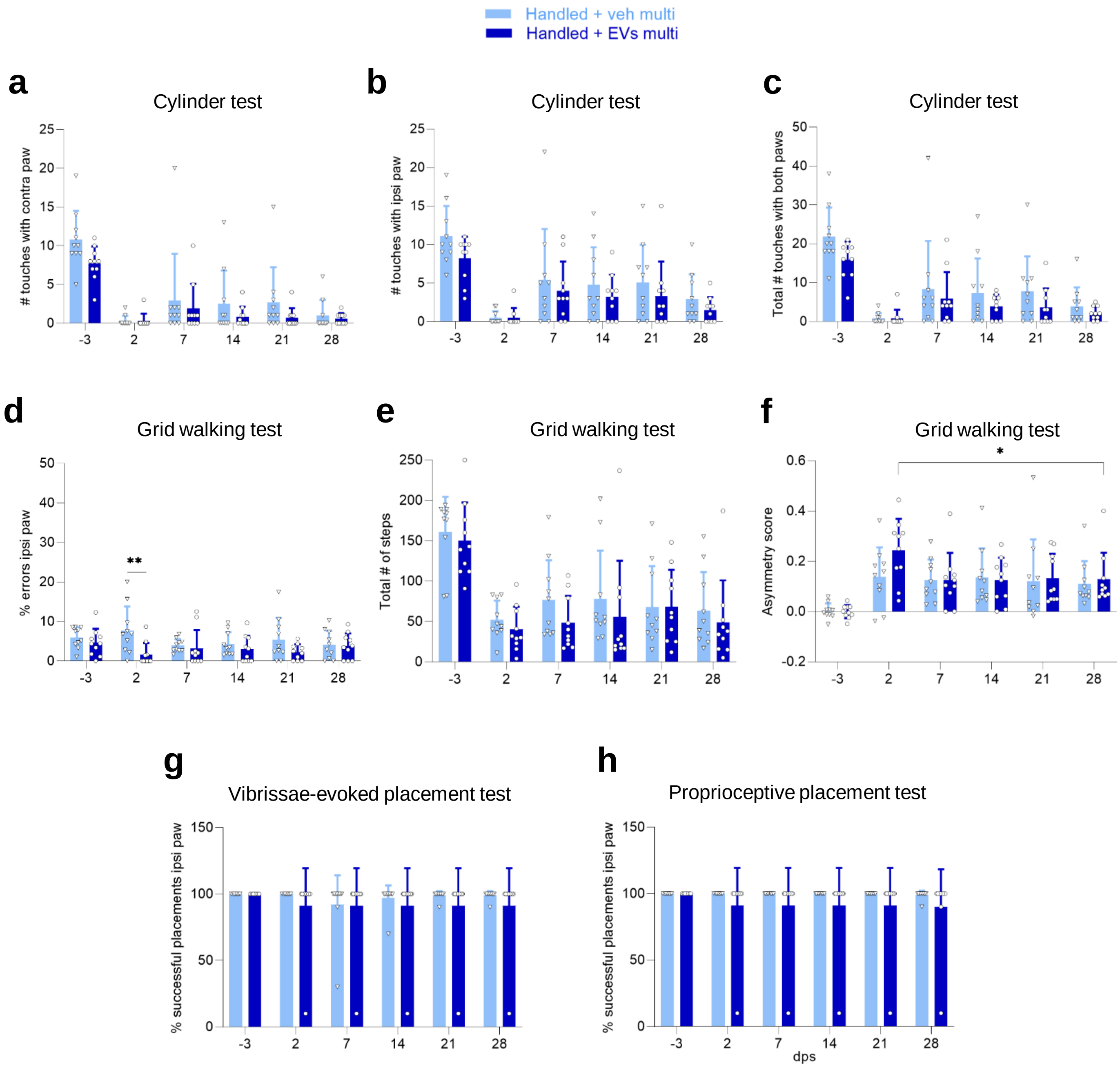

Figure S3. Handled rats treated with multidose EVs do not recover in the cylinder, grid walking, or forelimb placing tests at 28 dps.

(a–h) Handled rats treated with a multidose of vehicle or EVs were tested at -3, 2, 7, 14, 21, and 28 dps on **(a)** the number of touches with the contralateral paw in the cylinder test, **(b)** the number of touches with the ipsilateral paw in the cylinder test, **(c)** the total number of touches with both paws in the cylinder test, **(d)** the percentage of errors with the ipsilateral foot in the walking grid test, **(e)** the total number of steps in the walking grid test, **(f)** the asymmetry score in the walking grid test, **(g)** the percentage of successful placements with the ipsilateral paw in the vibrissae-evoked forelimb placing test, and **(h)** the percentage of successful placements with the ipsilateral paw in the proprioceptive forelimb placing test.

All data was analyzed by two-way repeated measures ANOVA followed by the post hoc Holm-Sidak's multiple comparisons test (*$p<0.05$, **$p<0.005$). Bars show mean +/− SD. Each symbol represents a single rat: handled + veh multi (*n=10*), handled + EVs multi (*n=10*).

Figure S4. Handled rats treated with a single dose of EVs do not recover in cylinder, grid walking, or forelimb placing tests at 28 dps

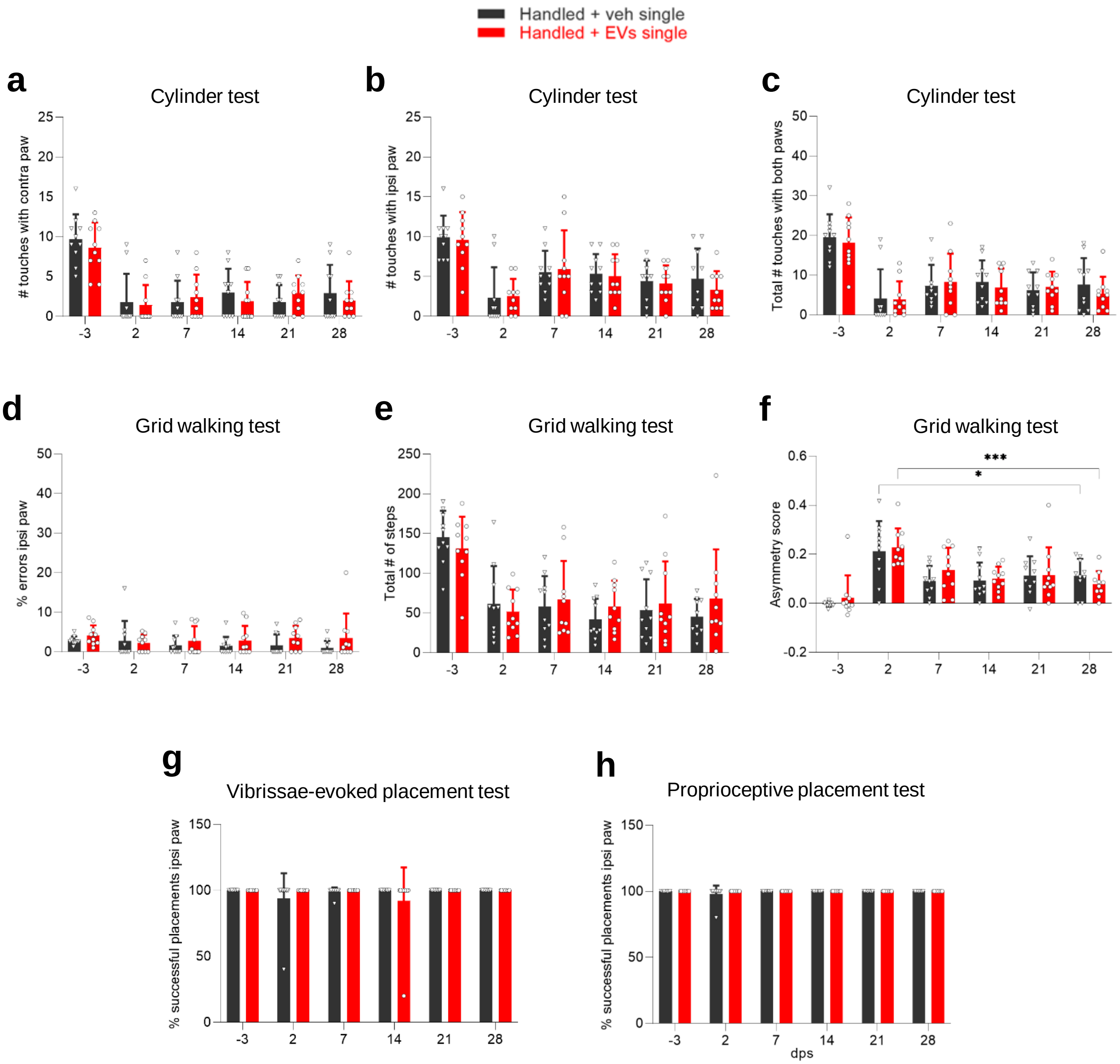

Figure S4. Handled rats treated with a single dose of EVs do not recover in cylinder, grid walking, or forelimb placing tests at 28 dps.

(a–h) Handled rats treated with a single dose of vehicle or EVs were tested at -3, 2, 7, 14, 21, and 28 dps on **(a)** the number of touches with the contralateral paw in the cylinder test, **(b)** the number of touches with the ipsilateral paw in the cylinder test, **(c)** the total number of touches with both paws in the cylinder test, **(d)** the percentage of errors with the ipsilateral foot in the walking grid test, **(e)** the total number of steps in the walking grid test, **(f)** the asymmetry score in the walking grid test, **(g)** the percentage of successful placements with the ipsilateral paw in the vibrissae-evoked forelimb placing test, and **(h)** the percentage of successful placements with the ipsilateral paw in the proprioceptive forelimb placing test.

All data was analyzed by two-way repeated measures ANOVA followed by the post hoc Holm-Sidak's multiple comparisons test (* $p<0.05$, *** $p<0.0005$). Bars show mean +/− SD. Each symbol represents a single rat: handled + veh single (*n=10*), handled + EVs single (*n=10*).

Figure S5. Handled rats treated with a single dose of EVs do not recover in the mNSS at 28 dps compared to handled rats treated with multidose

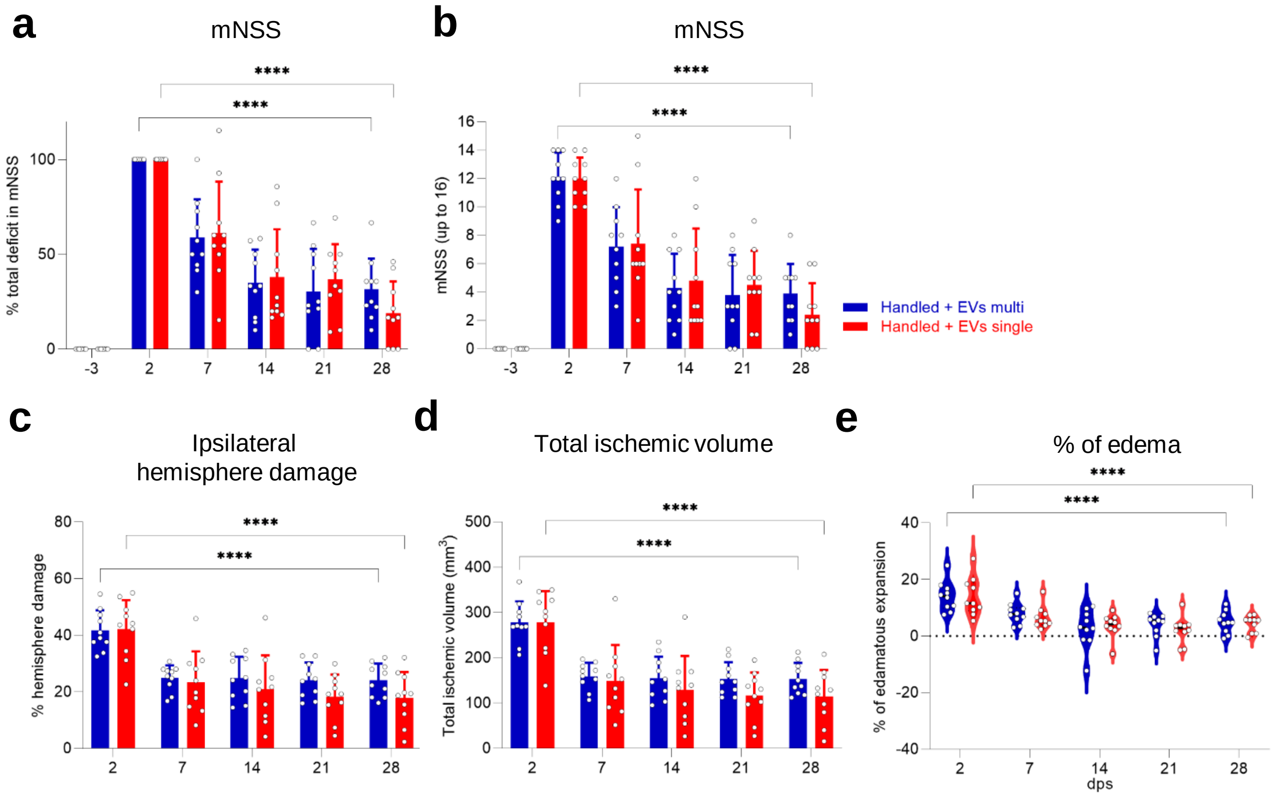

Figure S5. Handled rats treated with a single dose of EVs do not recover in the mNSS at 28 dps compared to handled rats treated with multidose.
(a) Functional recovery in handled rats receiving a single or multidose of EVs after MCAO stroke, shown as a percentage in total deficit in the modified Neurological Severity Score (mNSS) at -3, 7, 14, 21, and 28 dps after normalizing the score of each animal to its score at 2 dps.
(b) Same data as (a), showing raw mNSS results before normalization at -3, 2, 7, 14, 21, and 28 dps.
(c) Quantification of the percentage of ipsilateral hemisphere damage using MR images at 2, 7, 14, 21, and 28 dps of handled rats treated with a single or multidose of EVs after MCAO stroke.
(d) Same data as (c), showing total ischemic volume.
(e) Same data as (c), showing percentage of edematous expansion of the ipsilateral hemisphere.
All data was analyzed by two-way repeated measures ANOVA followed by the post hoc Holm-Sidak's multiple comparisons test (****$p<0.0001$). Bars show mean +/− SD. Each symbol represents a single rat: handled + EVs multi (*n=10*), handled + EVs single (*n=10*).
