# Supplementary material for: Recovery after human bone marrow mesenchymal stem cells (hBM-MSCs)-derived extracellular vesicles (EVs) treatment in post-MCAO rats requires repeated handling
Source: PLoS One. 2024 Oct 21;19(10):e0312298. doi: 10.1371/journal.pone.0312298 (PMC11493303; doi:10.1371/journal.pone.0312298)
Supplement: S1 Raw image — (PDF) [file pone.0312298.s003.pdf]

# S1 RAW IMAGES

Recovery after human bone marrow mesenchymal stem cells (hBM-MSCs)-derived extracellular vesicles (EVs) treatment in post-MCAO rats requires repeated handling.

# Original uncropped blots from Fig 1b.

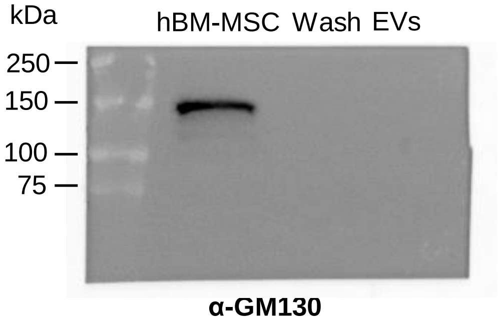


**α-GM130**

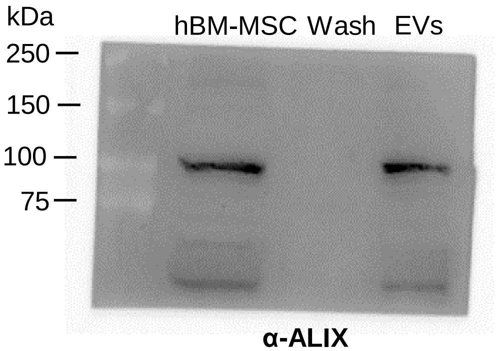


**α-ALIX**

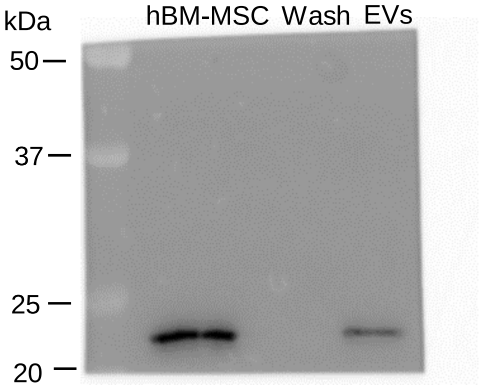


**α-CD9**

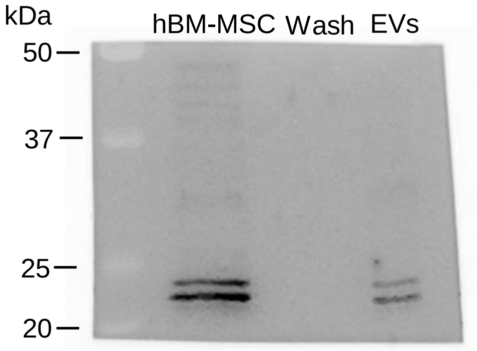


**α-CD81**

# Original uncropped TEM image from Fig 1c.

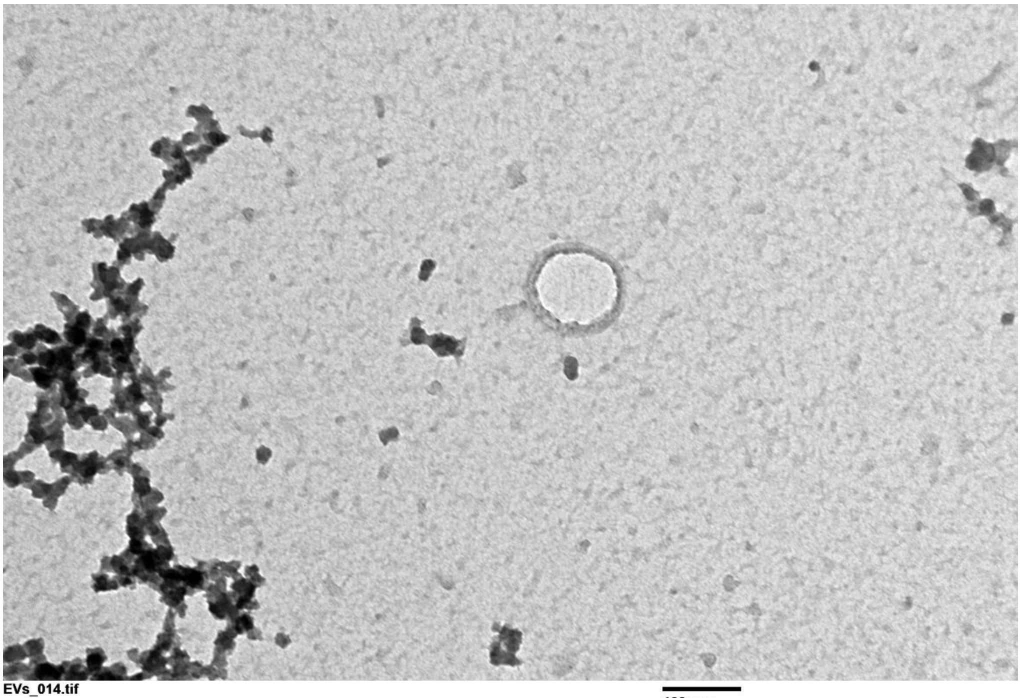

EVs_014.tif

Cal: 0.000432 µm/pix
10:50 2024-06-17

Camera: BIOSPR12, Exposure: 2400 (ms) x 1 std. frames, Gain: 2, Bin: 1
Gamma: 1.00, No Sharpening, Normal Contrast

100 nm
HV=80kV
Direct Mag: 42000 x
AMT Camera System
