# Supplementary material for: Recovery after human bone marrow mesenchymal stem cells (hBM-MSCs)-derived extracellular vesicles (EVs) treatment in post-MCAO rats requires repeated handling
Source: PLoS One. 2024 Oct 21;19(10):e0312298. doi: 10.1371/journal.pone.0312298 (PMC11493303; doi:10.1371/journal.pone.0312298)
Supplement: Attachment — Submitted filename: Response to reviewers_PLOS One 2024_Gomez Galvez et al 2024.pdf [file pone.0312298.s005.pdf]

June 19, 2024

Dear Dr. Hansen Chen,

Please find enclosed our revised manuscript “Recovery after human bone marrow mesenchymal stem cells (hBM-MSCs)-derived extracellular vesicles (EVs) treatment in post-MCAO rats requires handling associated with repeated behavioral testing” (PONE-D-24-14380). We are incredibly grateful to the editor and reviewers for their insightful and constructive comments, and we have addressed each point raised as completely as possible.

Additional note to the editor: **there was a typo in our title** (“hMB-MSCs” instead of “hBM-MSCs”). **This spelling error has been corrected** in the main manuscript and supplementary documents.

***

Journal Requirements

1. Please ensure that your manuscript meets PLOS ONE's style requirements, including those for file naming.

To the best of our knowledge, our manuscript meets PLOS ONE's style requirements now.

2. To comply with PLOS ONE submissions requirements, in your Methods section, please provide additional information regarding the experiments involving animals and ensure you have included details on (1) methods of sacrifice, (2) methods of anesthesia and/or analgesia, and (3) efforts to alleviate suffering.

We appreciate and agree with the comment about rigorously reporting detailed methods, especially those involving animals. The manuscript now includes all these points: (1) methods of sacrifice (line 347), (2) methods of anesthesia and/or analgesia (lines 152,174,345), (3) efforts to alleviate suffering (lines 123, 174, 176-178). Please let us know if this is insufficient, and we will add more details to the Methods.

3. Please complete your Competing Interests on the online submission form to state any Competing Interests. This information should be included in your cover letter; we will change the online submission form on your behalf.

Competing interests were included in our cover letter.

4. PLOS ONE now requires that authors provide the original uncropped and unadjusted images underlying all blot or gel results reported in a submission’s Figs or Supporting Information files.

Uncropped blots have been added to **Supporting File 4: S1_raw_images**.

5. We note that Fig 1 in your submission contains copyrighted image. We require you to either (1) present written permission from the copyright holder to publish these Figs specifically under the CC BY 4.0 license, or (2) remove the Figs from your submission.3

Original diagrams created with BioRender (**Figs 1d and S1a**) have been removed and substituted with others without copyright. Text in Acknowledgments was also modified to clarify this: "Diagrams in **Figs 1d** and **S1a** were created using Adobe InDesign (RRID:SCR_021799)." (line 666).

Editor Comments

1. Please specify the number of rats housed per cage in the Methods section.

The number of rats housed per cage is now specified in the Methods section (line 127).

2. Clarify whether the rats were randomized when assigned to groups, and indicate whether the surgeons and investigators conducting behavioral tests were blinded to the treatments.

As suggested, more clarification has been added to the randomization (line 134) and blinding (lines 183-185) part of this study in the Methods section.

3. In the Methods section, it is mentioned that the rats received 20ml of warm saline. Could you specify whether this was administered via intraperitoneal injection?

Rats received warm saline subcutaneously, and this has been added to the text (lines 175 and 177).

4. The repeated treatment group also involves repeated exposure to isoflurane, which might affect the recovery process. Please discuss this potential impact in your manuscript.

This is a very interesting and valid point previously demonstrated in multiple publications using the MCAO model in rats (Kim et al., 2015, PMID: 31024240; Wang et al., 2016, PMID: 26898453; Peng et al., 2019, PMID: 31024240). All our experimental groups include vehicle controls, except when "Non-handled + veh single" was compared to "Handled + veh single" (**Fig 2**). In this case, the "Handled" group received an extra ~15 min/day of exposure to isoflurane at 7, 14, and 21 dps due to MRIs. Our experimental animals received lower doses of isoflurane compared to other studies (e.g., 1h of isoflurane just after removing the filament and starting reperfusion in references) (Kim et al, 2015, PMID: 31024240; Wang et al, 2016, PMID: 26898453; Peng et al, 2019, PMID: 31024240). Thus, according to previous reports, we believe our short isoflurane exposure is insufficient to enhance recovery. As suggested by the reviewer, we acknowledge and discuss this possibility in the manuscript (lines 567-571).

Reviewer #1

The authors administered extracellular vesicles (EVs) derived from bone marrow mesenchymal stem cells (BM-MSCs) into the ischemic brain of MCAO models to enhance functional recovery. They evaluated the efficacy of single versus multiple doses using behavioral tests and assessed injury size over time. While some data supports their conclusions, additional information is needed before acceptance of the manuscript.

Major Concerns:

1. The MRI images do not correlate consistently with the quantification of brain damage depicted in Figs 2, 3, and 4.

We have replaced some of the MRI images in **Figs 2–5**. We have added a red dotted line to delineate the ischemic core. To clarify the MRI analysis, more details have been added to the methods section (lines 255-264) and Fig legends.

2. The ischemic brain typically experiences edema followed by chronic inflammation. Behavioral tests concluding on day 28 are insufficient to confirm functional recovery in the animals.

This is a valid concern especially because our treatments could have affected edema. There is a significant reduction in the % of the edematous expansion at 28 dps compared to 2 dps in all four groups analyzed, starting at 7 dps and stabilizing between 14 and 28 dps (**Figs S2b, S2d, S2f, and S2h; Table S5**). This suggests that all groups resolved the edema by 28 dps, independently of their treatment (handling or EVs). This, of course, affects functional recovery in the animals, but it does not explain the differences found between treatment groups. Additionally, the time point of 28 dps has been previously used in many other publications to assess functional recovery in the MCAO model (Otero-Ortega et al, 2020, PMID: 32075692; Venkat et al, 2020, PMID: 32889008; Dumbrava et al, 2022, PMID: 34757568).

3. How did EVs affect the damaged brain—by reducing inflammation or providing nutritional support? What differentiates single-dose from multi-dose treatments? The authors should explain the underlying mechanisms supporting their findings.

We acknowledge that it is very important for the field to understand the underlying cellular and molecular mechanisms that improve recovery with EVs. We have now added this discussion point to our manuscript (lines 595-599).

4. The authors should specify the number of animals used in behavioral tests, given the substantial standard deviation observed in Figs 3 and 4.

We thank the reviewer for the comment. As suggested, in addition to **Table S1** (see below), the number of rats used for the behavioral tests has been added to the Methods section (lines 136-138) and Fig legends (**Figs 2–5 and S1–S5**).

Table S1. Number of MCAO rats with treatments and behavioral assessments for each cohort.

Cohort	Intranasal treatment	Number of MCAO rats *(n)*	Behavior
**Non-handled**	Vehicle single dose	*n=12*	mNSS at 2, 28, 56 dps
	EVs single dose	*n=13*	mNSS at 2, 28, 56 dps
**Handled**	Vehicle single dose	*n=10*	mNSS, cylinder, corner, beam balance, grid walking forelimb placement at 2, 7, 14, 21, 28 dps
	EVs single dose	*n=10*	mNSS, cylinder, corner, beam balance, grid walking forelimb placement at 2, 7, 14, 21, 28 dps
**Handled**	Vehicle multidose	*n=10*	mNSS, cylinder, corner, beam balance, grid walking forelimb placement at 2, 7, 14, 21, 28 dps
	EVs multidose	*n=10*	mNSS, cylinder, corner, beam balance, grid walking forelimb placement at 2, 7, 14, 21, 28 dps

Minor Revisions:

1. In Fig 1b, molecular weight ladders are not included in the Western blot (WB) images.

We thank the reviewer for the comment. Molecular weight ladders have been included in **Fig 1b**.

Reviewer #2

This manuscript carefully investigates the effects of handling and/or intranasal EVs on the recovery after MCAO in rats, providing solid evidence that intensive behavior testing alone improves recovery after stroke, which could be enhanced after intranasal single dose of EVs. The authors have done rigorous experiments addressing this, while some concerns are needed to be addressed to fully clarify the statement.

Major points:

1. Fig. 2: effect of handling on neurological function after MCAO

The authors have shown that intensive behavior tests improve scores in mNSS as 28 dps. This is quite meaningful to the field since behavior tests have been typically used to assess performance after stroke and the contribution to stroke recovery has been overlooked. To further address the temporal effect of handling, it would be interesting to conduct these experiments at different timepoints, for example D7, D14, D21 dps like Fig. 3-4.

As sensorimotor tests are frequently used in preclinical stroke studies, and rehab is frequently given to humans after a stroke, we only focused on the handling component of the paper due to extensive behavior instead of pure spontaneous recovery without handling. We agree that it would be interesting to know what happens when only mNSS is measured at 7, *or* 14, *or* 21 dps in non-handled rats. In relation to this question, a previous publication mentioned in our manuscript has

shown spontaneous recovery after MCAO in rats when only exposed to the neurological score test (Heras-Romero et al., 2022, PMID: 34563674). In this study, neurological deficits decreased by 7 dps, progressively improving by 14 dps, and plateauing by 21 dps.

2. Fig. 3-4: effect of EVs (single and multiple doses) on neurological function after MCAO

The authors further explored whether intranasal EVs could enhance the handling-mediated neurological improvement after stroke at different time points, and found that single dose, instead of multiple doses showed synergistic effect, which might result from the lack of efficacy treated with multidose EVs according to the authors' discussion. It would be important to address some concerns to further support the current statement.

2.1. EVs presence in the brain? The authors have nicely observed the presence of EVs 6 hours after a single dose (2.4 x 10^9 vesicles). How about the time course? It would be interesting to see if these EVs persists in the brain since most improvement after a single dose were observed in D28 dps (Fig. 4a-c). In addition, does EVs present in brains from MCAO rats after the lower multiple doses (0.8 x 10^9 vesicles)? A lower presence of EVs may explain the undetectable effects in stroke (Fig. 3).

Our studies demonstrated that EVs administrated intranasally can reach the brain and the injury site. Tracking the time course distribution of EVs *in vivo* presented some important caveats. Using fluorescent dyes to label EVs (as we did) is a great challenge for the field due to the characteristics of many commercial fluorescent dyes. Dyes can induce aggregation of EVs, or the dye's half-life cannot be long enough to be detected after a certain time (Yi et al, 2020, PMID: 31963931). Additionally, other cells can phagocytose labeled EVs, or EVs can release the dye and then be taken by other cells, keeping the dye long-term and producing misleading results (Yi et al., 2020, PMID: 31963931; Aimaletdinov et al., 2022, PMID: 36232613). We did not perform a long-term study using labeled EVs for these reasons. Nevertheless, previous studies using gold nanoparticles to label the EVs and computed tomography (CT) (Betzer et al, 2017, PMID: 28960957; Perets et al, 2019, PMID: 30761901) have demonstrated that intranasally administrated EVs are retained for at least 96h in the brain of a mouse model of focal stroke (ETH1-1 injection). These gold-labeled EVs were found around the ischemic region, as we saw in our study. Interestingly, they also saw that healthy control brains cleared gold-labeled EVs 24h post-administration.

As the reviewer suggested, and as we discussed in lines 579-583, we speculate that the lack of efficiency of the multidose may be due to the low concentration of EVs per dose. Unfortunately, it is very difficult to assess if an accumulation of labeled EVs occurs in the peri-infarct area at 28 dps using the multidose for the characteristic of the dye explained above.

2.2. Comparison between single dose and multiple doses of EVs on neurological function after MCAO? The authors have done separate experiments to compare the effect of single (Fig. 4) or multiple doses (Fig. 3) of EVs to handling alone on neurological function. Considering the baseline

of most behavior tests may vary, it would be interesting to see how the dose of EVs may affect the handling-mediated neurological function in one set of experiment, or at least discusse in an integrated data from Fig. 3-4.

After integrating the data from **Figs 3 and 4**, comparing the “Handled + EVs multi” versus the “Handled + EVs single” (new **Fig S5**), no differences were found between these two groups in the mNSS at 28 dps (**Figs S5a,b**), percentage of ipsilateral hemisphere damage (**Fig S5c**), total ischemic volume (**Fig S5d**), and edema (**Fig S5e**) at 28 dps. This corroborates our modest improvement with the single dose (**Fig 4a**). It also emphasizes that the main recovery is largely due to handling. These findings have been added to the Results section (line 489) and Discussion (line 575).

2.3. Statistics analysis of EVs on neurological function after MCAO? The authors have conducted 2-way ANOVA to include both time and treatment (veh or EVs) to assess if there is a significant improvement, and nicely provided the raw data for the comparison in all individual days in the supplement files. However, it would be necessary to include the P value for a row and column factor (time and treatment here) in a 2-way ANOVA test. This would be more relevant to suggest if an overall improvement exist over time and/or after EVs treatment, in addition to the improvement in specific days.

We thank the reviewer for bringing up this important point. Results for each ANOVA test have been added to **Table S5**.

3. MRI analysis on the volume of the injury or edema

3.1. The authors have shown that either handling or EVs treatment had no effect on changes in injury volume after MCAO in rats, in contrast to the neurological function (Fig. 2-5). In Fig. 2c-d, less ipsilateral hemisphere damages were observed at D28 dps, while the representative images didn’t show a less bright volume. It would be necessary to clarify how the damage area were calculated in the Methods, and make sure to include representative images. Similar in Fig. 3-5.

See response to Reviewer #1 Point 1. For further clarification, we did not quantify the damage by measuring the brightness but by calculating the total volume after profiling the contour of the ischemic core in each coronal section.

Minor points:

1. Cohorts and experimental groups in Methods:

The authors described that three different cohorts were established (line 133-135). In fact, the authors have done 5 groups rigorously to evaluate the effects of both handling and EVs treatment on stroke recovery (Table S1). It would be nice to also include the group information in Methods to make it easier to follow.

Number of rats used for the behavioral tests has been added to the Methods section (lines 136-138) and Fig legends (**Figs 2–4 and S1–S4**).

Reviewer #3

This is a well written manuscript, I feel comfortable reading it. However, there are some major concerns about the Figs and the statistical analysis.

1. Fig 1B. The western blot should include the markers showing the MW of the band.

We thank the reviewer for the comment. Molecular weight ladders have been included in **Fig 1b**.

2. Fig 1E. The Hoechest staining seems to be out of focus in the contralateral image, the authors needs a better image and do the quantification of the percentage of ExoGlow+ cells.

We have now included quantifications of the average size of ExoGlow+ particles in both hemispheres. Methods (lines 360-371, 374), Results (line 393), and **Fig 1f** have been updated. New fluorescence images have been added to **Fig 1e**.

3. Fig 2D. In the test, the authors wrote "No differences were found between handled and non-handled rats in the percentage of ipsilateral hemisphere damage." The point is "no difference". Therefore, the authors should compare "handled" vs "non-handled", not "2dps" vs "28dps" in quantification.

We apologize if this was not clear. All the raw data for the post-hoc tests for time and treatment are included in **Table S5**. We have also included the p-value for the row (time) and column (treatment) factors in **Table S5.** Comparison between handled and non-handled is mentioned in lines 422-424, and shown in **Fig 2** and **Table S5**.

4. Fig 2D. The authors should discuss why handling improve neurological function, but not the ipsilateral hemisphere damage. What are the link between these two tests and what could be the potential explanation that two assays shows different results.

Traditionally, a general idea in the stroke field has been that a reduction of ischemic damage was needed to obtain sensorimotor recovery. Most of these results and conclusions were based on macroscopy-histologic examination of the tissue, using techniques such as TTC (2,3,5-triphenyl tetrazolium chloride) or H&E (hematoxylin and eosin) staining, which do not allow *in vivo* longitudinal examinations of the ischemic core from the same animal. In our experience, and due to the intrinsic variability of each MCAO stroke between animals, an MRI allows a better follow-up of the injury progression in a single animal. It allows better inclusion criteria, where only animals with similar strokes are included in the study.

To add clarity about these results and conclusions, we have added more details to the previous discussion (lines 636-652).

5. Fig 3. The authors are comparing handling vs handling+multi EVs in this Fig, but in every panel the authors labels significant difference between "2dps and 28dps", but not "handling vs handling+multi EVs" this is quite misleading to readers.

For transparency, we included all the raw data from our statistical analyses (t-test, 2-way ANOVA and post-hoc tests) in **Table S5**. We performed all the statistical analyses to see if animals recovered over time and/or after EVs treatment. For clarity, our graphs did not show “non-significant” results for all post-hoc comparisons (time and treatment). Additionally, we only showed changes between the beginning of the treatment (2 dps) and the endpoint (28 dps) in the graphs to simplify the representation of data. To note, we performed other statistics to check possible improvement on specific days, and these results are shown in **Table S5**.

***

We hope that with these revisions, the manuscript will now be found acceptable for publication in PLOS One.

Please do not hesitate to contact us if we can further assist you.

Sincerely,

Elena Blanco-Suarez, PhD

Lorraine Iacovitti, PhD
