# Supplementary material for: Recovery after human bone marrow mesenchymal stem cells (hBM-MSCs)-derived extracellular vesicles (EVs) treatment in post-MCAO rats requires repeated handling
Source: PLoS One. 2024 Oct 21;19(10):e0312298. doi: 10.1371/journal.pone.0312298 (PMC11493303; doi:10.1371/journal.pone.0312298)
Supplement: Attachment — Submitted filename: Review comments_20240504.docx [file pone.0312298.s004.docx]

This manuscript carefully investigates the effects of handling and/or intranasal EVs on the recovery after MCAO in rats, providing solid evidence that intensive behavior testing alone improves recovery after stroke, which could be enhanced after intranasal single dose of EVs. The authors have done rigorous experiments addressing this, while some concerns are needed to be addressed to fully clarify the statement.

Major points:

1. Fig. 2: effect of handling on neurological function after MCAO

The authors have shown that intensive behavior tests improve scores in mNSS as 28 dps. This is quite meaningful to the field since behavior tests have been typically used to assess performance after stroke and the contribution to stroke recovery has been overlooked. To further address the temporal effect of handling, it would be interesting to conduct these experiments at different timepoints, for example D7, D14, D21 dps like Fig. 3-4.

2. Fig. 3-4: effect of EVs (single and multiple doses) on neurological function after MCAO

The authors further explored whether intranasal EVs could enhance the handling-mediated neurological improvement after stroke at different time points, and found that single dose, instead of multiple doses showed synergistic effect, which might result from the lack of efficacy treated with multidose EVs according to the authors’ discussion. It would be important to address some concerns to further support the current statement.

EVs presence in the brain? The authors have nicely observed the presence of EVs 6 hours after a single dose (2.4 x 10^9 vesicles). How about the time course? It would be interesting to see if these EVs persists in the brain since most improvement after a single dose were observed in D28 dps (Fig. 4a-c). In addition, does EVs present in brains from MCAO rats after the lower multiple doses (0.8 x 10^9 vesicles)? A lower presence of EVs may explain the undetectable effects in stroke (Fig. 3).

Comparison between single dose and multiple doses of EVs on neurological function after MCAO? The authors have done separate experiments to compare the effect of single (Fig. 4) or multiple doses (Fig. 3) of EVs to handling alone on neurological function. Considering the baseline of most behavior tests may vary, it would be interesting to see how the dose of EVs may affect the handling-mediated neurological function in one set of experiment, or at least discusse in an integrated data from Fig. 3-4.

Statistics analysis of EVs on neurological function after MCAO? The authors have conducted 2-way ANOVA to include both time and treatment (veh or EVs) to assess if there is a significant improvement, and nicely provided the raw data for the comparison in all individual days in the supplement files. However, it would be necessary to include the P value for a row and column factor (time and treatment here) in a 2-way ANOVA test. This would be more relevant to suggest if an overall improvement exist over time and/or after EVs treatment, in addition to the improvement in specific days.

3. MRI analysis on the volume of the injury or edema

The authors have shown that either handling or EVs treatment had no effect on changes in injury volume after MCAO in rats, in contrast to the neurological function (Fig. 2-5). In Fig. 2c-d, less ipsilateral hemisphere damages were observed at D28 dps, while the representative images didn’t show a less bright volume. It would be necessary to clarify how the damage area were calculated in the Methods, and make sure to include representative images. Similar in Fig. 3-5.

Minor points:

1. Cohorts and experimental groups in Methods:

The authors described that three different cohorts were established (line 133-135). In fact, the authors have done 5 groups rigorously to evaluate the effects of both handling and EVs treatment on stroke recovery (Table S1). It would be nice to also include the group information in Methods to make it easier to follow.
