# Supplementary material for: Recovery after human bone marrow mesenchymal stem cells (hBM-MSCs)-derived extracellular vesicles (EVs) treatment in post-MCAO rats requires repeated handling
Source: PLoS One. 2024 Oct 21;19(10):e0312298. doi: 10.1371/journal.pone.0312298 (PMC11493303; doi:10.1371/journal.pone.0312298)
Supplement: Attachment — Submitted filename: Response to reviewers_Gomez Galvez et al 2024.docx [file pone.0312298.s006.docx]

September 12, 2024

Dear Dr. Hansen Chen,

Please find enclosed our second revision for the manuscript “Recovery after human bone marrow mesenchymal stem cells (hBM-MSCs)-derived extracellular vesicles (EVs) treatment in post-MCAO rats requires repeated handling” (PONE-D-24-14380). We are grateful to the Reviewers for their insightful and constructive comments, and we have addressed each point raised as indicated below. The manuscript and supplementary documents have been modified accordingly.

Additional note to the editor: **the title has been changed as suggested by Reviewer #1**.

***

**Journal Requirements**

1. Please review your reference list to ensure that it is complete and correct. If you have cited papers that have been retracted, please include the rationale for doing so in the manuscript text, or remove these references and replace them with relevant current references. Any changes to the reference list should be mentioned in the rebuttal letter that accompanies your revised manuscript. If you need to cite a retracted article, indicate the article’s retracted status in the References list and also include a citation and full reference for the retraction notice.

To the best of our knowledge, our reference list is complete and correct. No papers have been retracted. Changes have been made to the reference list after adding more articles to the Discussion as suggested by Reviewer #2.

**Editor Comment**

1. Please address the reviewers' comments for the minor revisions. If you prefer the current title, you are not required to change it.

Minor revisions have been addressed. The title was changed following Reviewer #1’s suggestion.

**Reviewer #1**

The authors developed a treatment based on hBM-MSCs-derived extracellular vesicles to reduce the damage size after MCAO. The revised manuscript is well done. The data fully support their view. I recommend removing "behavioral testing" from the title.

As suggested by the Reviewer, “behavioral testing” has been removed from the title.

**Reviewer #2**

Minor points:

1. mNSS at D7, D14, D21 dps in handled rats

The authors have shown that intensive behavior tests improve scores in mNSS as 28 dps. To further address the temporal effect of handling, it would be interesting to measure mNSS at different timepoints, for example D7, D14, D21 dps. Alternatively, please include previous studies in Discussion.

We thank the Reviewer for the comment. In our study, we observed that handled rats started their recovery in the mNSS at 7 dps, plateauing around 14 or 21 dps depending on the group (**Fig 3** and **Fig 4**, **Table S5**). These results have been added to the manuscript for clarity (lines 446, 477, 557 in unmarked manuscript). As suggested, we added previous studies in our Discussion (lines 557-561 in unmarked manuscript) to explain how the lack of handling affects the mNSS at 7, 14, or 21 dps.

2. Please discuss the presence of EVs in the brain

The author did not perform a long-term study using labeled EVs for technical limitations, and provided previous studies to support the likely presence of EVs in the brain (Betzer et al, 2017, PMID: 28960957; Perets et al, 2019, PMID: 30761901). It would be necessary to include this presumption in Discussion.

As suggested by the Reviewer, we added this part to the Discussion (lines 617-626 in unmarked manuscript).

**Reviewer #3**

The abstract is too long in the current version. The authors described too much detail in the abstract. In the last sentence of the manuscript: These results show the importance of rehabilitation in combination with other treatments and highlight how intensive behavioral testing might influence functional recovery after stroke, especially when other treatments are also given. Here, "other treatments" seems to general, this kind of sentence could be present in discussion, but is quite in appropriate in abstract.

We appreciate the Reviewer's suggestions. The abstract has been made shorter and more clarity has been added to the conclusion in the abstract: “These results show the importance of rehabilitation in combination with other treatments such as EVs, and highlight how extensive behavioral testing might influence functional recovery after stroke” (line 47 in unmarked manuscript).

The authors only showed handling+single EV can improve mNSS and Beam Balance Test, the difference is mild. Therefore, the effect of EV is not solid, and cannot be concluded scientifically.

We agree with the Reviewer’s comment. We have added more clarity throughout the manuscript to not overstate our results (lines: 478, 480, 484, 492, 546, 570, 575, 627 in unmarked manuscript).

***

We hope that with these minor revisions, the manuscript will now be found acceptable for publication in PLOS One.

Please do not hesitate to contact us if we can further assist you.

Sincerely,

Elena Blanco-Suarez, PhD

Lorraine Iacovitti, PhD
